# Reproduction Modes and Conservation Implications in Three Polyploid *Sorbus* Stenoendemics in Eastern Slovakia (Central Europe)

**DOI:** 10.3390/plants12020373

**Published:** 2023-01-13

**Authors:** Vladislav Kolarčik, Mária Mirková, Vlastimil Mikoláš

**Affiliations:** 1 Institute of Biology and Ecology, Faculty of Science, Pavol Jozef Šafárik University, Mánesova 23, SK-041 54 Košice, Slovakia; 2 Independent Researcher, Hanojská 4, SK-040 13 Košice, Slovakia

**Keywords:** apomixis, Central Europe, endemism, fertilization, flow cytometry, hybridization, parthenogenesis, pollen stainability, polyploidy, pseudogamy

## Abstract

The remarkable species diversity of the genus *Sorbus* is a result of polyploidization and frequent hybridization between interacting species of different cytotypes. Moreover, hybridization is possible between several parental taxa. Gametophytic apomixis, which is common among polyploid *Sorbus* taxa, indicates the role of clonal reproduction in the evolutionary stabilization of hybridogeneous genotypes. The precise determination of the origin of seeds and their quantitative evaluation may elucidate inter-cytotype interactions, the potential role of mixed-cytotype populations in evolutionary success, and the long-term survival of some hybrid species. We investigated the reproduction modes of selected species of *Sorbus* in mixed-cytotype populations in eastern Slovakia, Central Europe. We determined the pollen quality, seed production rate, and the ploidy level of mature trees, as well as the origin of the embryo and endosperm in seeds of the stenoendemics *S. amici-petri*, *S. dolomiticola*, and *S. hornadensis*. The tetraploids *S. amici-petri* and *S. hornadensis* are characterized by regular and highly stainable pollen grains and reproduce predominantly via pseudogamous apomixis. In contrast, triploid *S. dolomiticola* usually has oval, heterogenous, and weakly stainable pollen grains, suggesting male meiotic irregularities. Although seeds originate via pseudogamous apomixis in *S. dolomiticola* as well, the ploidy level of sperm cells participating in the fertilization of central cells is usually determined by co-occurring species of different cytotypes. This suggests that maintaining mating partners is necessary for the long-term survival of a triploid species. We documented rare B_III_ hybrids and the residual sexuality in tetraploids. The distribution of seeds of meiotic and apomeiotic origins in *S. amici-petri* shows bimodal characteristics; however, genotypes with predominantly sexual seed types are rare. Reproduction modes documented in polyploid stenoendemics of *Sorbus* and inferred microevolutionary intercytotype relationships highlight the mixed-cytotype populations as the source of biodiversity in apomictic plant complexes. We suggest that conservation efforts should focus on maintaining the species and cytotypic diversity of *Sorbus* populations, especially when it comes to the conservation of triploid species.

## 1. Introduction

Reproduction is a trait of organisms that involves the development of male and female gametes and the origin of new individuals [1,2]. Additionally, it plays an important role in the diversification of plant groups and their subsequent evolution [3]. Plants can reproduce sexually, asexually, or hemisexually [4,5], and can even utilize combinations of these strategies; for instance, some offspring of an individual may originate sexually and others asexually [6]. Sexual reproduction produces new individuals with genetic features inherited from both parents. Asexual reproduction gives rise to clonal populations, a group of individuals that are genetically identical to their maternal or paternal individual [7,8,9]. Clones may originate vegetatively or through seeds (agamospermy) [10,11]. Hemisexuality is rather rare and involves the inheritance of part of the genetic material sexually and the other part clonally [5,12]. Plant populations or lineages may exhibit various modes of reproduction, which are reflected in their genetic, phytochemical, and morphological diversity [13,14,15]. Therefore, determining the reproduction mode may be crucial for understanding population and species microevolutionary dynamics. This is particularly important in the case of endemic, vulnerable, and threatened plants, and the knowledge of reproduction modes may even help to improve conservation planning and management.

The genus *Sorbus* L. represents a complex, taxonomically difficult group of plants [16]. Species differentiation in *Sorbus* is driven by polyploidization and hybridization, coupled with a common cytotype-specific reproductive segregation (diploids are sexual and polyploids are predominantly asexual) [16,17,18,19,20,21,22,23]. Reproductive systems in *Sorbus* are well understood. Several studies have shown that asexual reproduction in *Sorbus* is characterized by the presence of gametophytic apomixis, the parthenogenetic development of embryos from unreduced egg cells, and nutritive tissue endosperm produced via pseudogamy (fertilization of unreduced central cells) [18,20,24,25,26]. The frequent sympatry of diploids and polyploids facilitates the origin of hybridogeneous genotypes, which may become fixed through the development of apomictic traits [27] (the emergence of apomeiosis/apospory, parthenogenesis, pseudogamy). Recently, detailed biosystematic investigations of the genus revealed several new apomictic species in Bosnia and Herzegovina, the Czech Republic, Hungary, and Sweden [19,28,29,30,31,32], indicating the prevalence of high evolutionary dynamics in this genus. Further investigations in new regions may reveal similar diploid-polyploid (sexual-apomictic) patterns and document new species.

**Figure 1:**
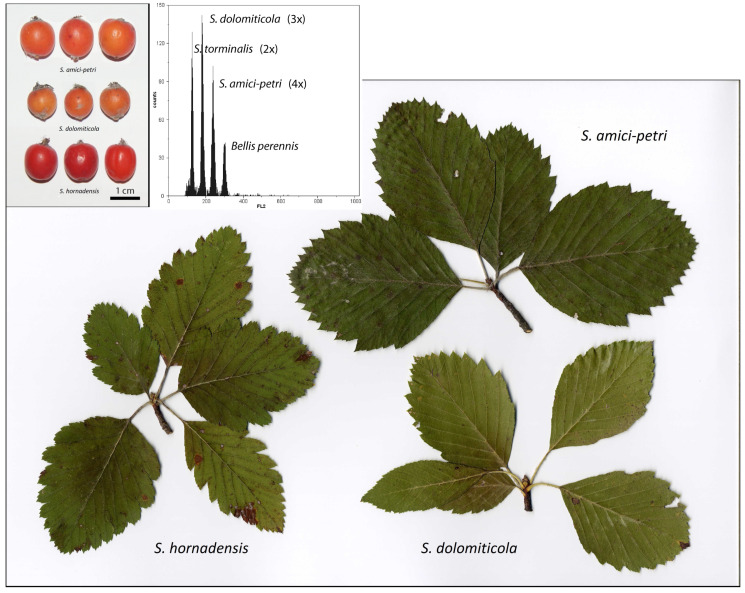
Three stenoendemic (taxon having very restricted distribution range) *Sorbus* species growing in eastern Slovakia (Central Europe) investigated in the present study. Leaves of short, sterile brachyblast and fruits (left inset) enabled precise identification of the species in the field. Example of flow cytometry (FCM) histogram (right inset) documenting variation in DNA content (relative fluorescence on *x*-axis) among different cytotypes: diploid *S. torminalis*, triploid *S. dolomiticola*, and tetraploid *S. amici-petri*. In FCM analyses, *Bellis perennis* L. was used as an internal reference standard. Note, *S. hornadensis* is also tetraploid.

Common co-occurrences of the diploid sexual species, *S. aria* (L.) Crantz, *S. aucuparia* L., and *S. torminalis* (L.) Crantz [20,22], as well as of other polyploids, presumably apomictic taxa, such as *S. danubialis* (Jáv.) Prodan (s.l.) and *S. thaiszii* (Soó) Kárpáti (s.l.) [20,33], may contribute to ongoing hybridizations and the emergence of new species in the Stredné Pohornádie valley in eastern Slovakia. In 1996–2015, V. Mikoláš described three species, *S. dolomiticola* Mikoláš, *S. amici-petri* Mikoláš, and *S. hornadensis* Mikoláš, which are narrow endemics of the region [34,35,36], and all of them are considered endangered [37]. The first species described from the region, *S. dolomiticola*, is a triploid, stenotopical species (able to tolerate only a restricted range of habitats or ecological conditions) growing in forest-steppe habitats on xerothermous steep slopes that is presumably a hybrid of *S. torminalis* and *S. danubialis* s.l. *Sorbus dolomiticola* is characterized by pale gray/white tomentose lower side of broadly ovate to nearly rhombic leaves and orange-red globose fruits that are 10.5–12.5 × 10.5–12.5 mm in size (Figure 1). *Sorbus amici-petri* is a tetraploid species that is supposedly a hybrid of *S. torminalis* and *S. thaiszii* s.l. It is similar to *S. dolomiticola* and is characterized by leaves that are broadly ovate to rhombic, but differs slightly in size, and has larger red (orange) globose fruits (11.0–13.0 × 13.0–14.0 mm). Such leaf and fruit features may be associated with a higher ploidy level in *S. amici-petri*. The species *S. hornadensis* is tetraploid and morphologically distinct from *S. dolomiticola* and *S. amici-petri*, and has supposedly evolved via hybridization between *S. thaiszii* s.l. and *S. aucuparia*. The species has broadly ovate, shallowly lobed leaves (usually five–seven pairs of lobes) and dark red, shortly cylindric fruits that are 11.0–13.5 × 10–12.5 mm in size. All three species and their putative parental taxa grow sympatrically in a few sites, such as in S-W oriented slopes near Kysak, Obišovce, and Trebejov villages in Stredné Pohornádie valley. These species probably reproduce asexually, and would likely be aposporous pseudogamous apomicts; however, this remains unverified to date.

In the present study, we document the modes of reproduction of three endemic species, *S. dolomiticola*, *S. amici-petri*, and *S. hornadensis*. We aimed to (i) assess reproduction success, i.e., regular seed formation; (ii) determine the frequency of presumed apomeiosis and, if present, rare meiosis; (iii) reconstruct the endosperm origin and infer whether intercytotype interaction is necessary for regular seed formation; and (iv) test for differences in reproduction modes between taxa in this study, e.g., variation in the frequency of meiosis, pseudogamy, and the fertilization of egg cells (possibly including the origin of B_III_ hybrids). We discuss the microevolutionary consequences of these reproduction modes and their potential threats to endemic species. Previous studies have shown that the pseudogamic origin of functional endosperms in the seeds of self-incompatible species may have important conservation implications [17,38].

## 2. Results

### 2.1. Flow Cytometric Determination of Ploidy Level of Mature Trees

The flow cytometry ploidy level measurements fully correspond to the morphology of each species. All of the trees of *S. amici-petri* were tetraploid (15 trees), those of *S. dolomiticola* were triploid (15 trees), and those of *S. hornadensis* were tetraploid (12 trees). Additionally, ploidy levels of supposedly diploid *S. aria* (4 trees) and *S. torminalis* (3 trees) were confirmed.

### 2.2. Pollen Stainability and Pollen Size

**Figure 2:**
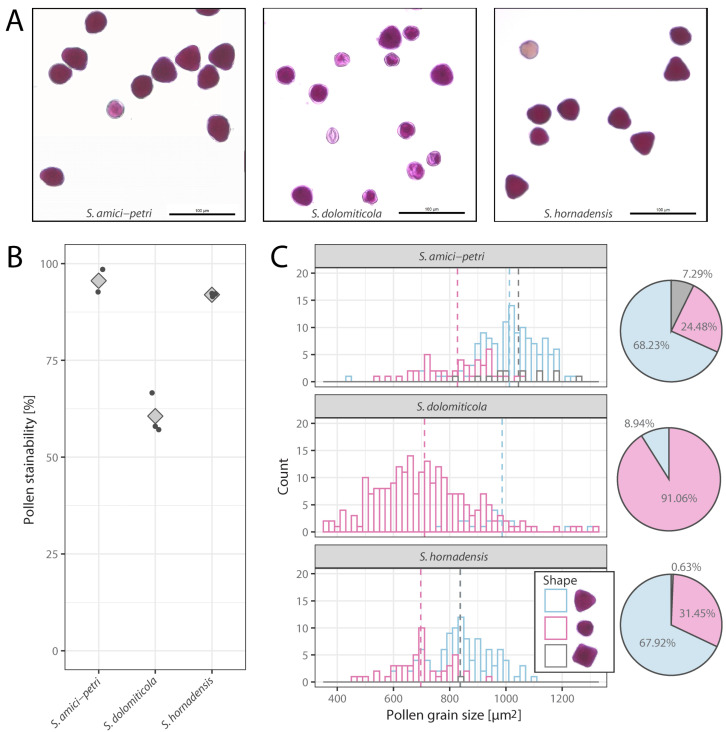
Pollen stainability and pollen size in polyploids *Sorbus amici-petri*, *S. dolomiticola*, and *S. hornadensis.* (**A**) Representative microscopic views of stained pollen grains of three species. (**B**) Variation in pollen stainability among species. (**C**) Pollen grain size and proportion of pollen shape types. In B, dots and diamonds represent pollen stainability per sample and their averages, respectively. Dashed lines are mean values in (**C**).

On average, pollen stainability was above 90% in tetraploids, 95.6% in *S. amici-petri*, and 91.9% in *S. hornadensis*. On average, triploid species *S. dolomiticola* showed a 60.6% decrease in pollen stainability (Figure 2). Stained pollen grains were mostly triangular; however, oval pollen grains were quite frequent, and a rare quadrangular pollen shape was also recorded in both tetraploids (Figure 2). In triploid *S. dolomiticola*, stained pollen grains were often oval, and triangular pollen grains were rare. Unstained pollen grains were usually irregular in shape and often shrunken. A size analysis of stained pollen grains showed that oval pollen grains of triploids were highly variable in size compared to those of tetraploids (Figure 2).

### 2.3. Seed Production Rate

**Figure 3:**
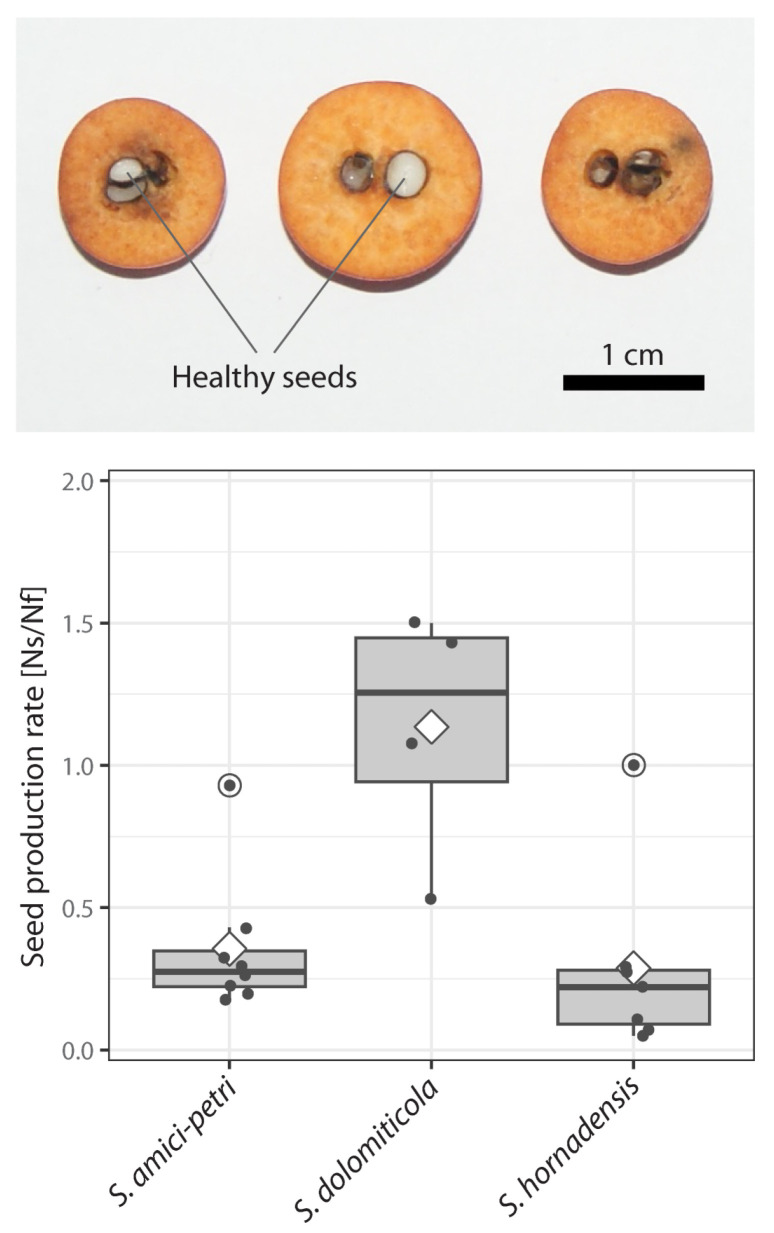
Seed production rate in polyploids *Sorbus amici-petri*, *S. dolomiticola*, and *S. hornadensis*. Examples of three halved fruits (*S. amici-petri*, basal halves) with well-developed seeds (upper subfigure). Variation in seed production rate (lower subfigure); dots represent seed production rate of individual trees; the lower and upper hinges of boxplot correspond to the first and third quartiles (delimit inter-quartile range; IQR), whiskers extend to ±1.5 × IQR, and empty circles represent outliers; horizontal lines and diamonds represent median and mean values, respectively. Ns–number of well-developed seeds, Nf–number of examined fruits.

Although all of the investigated individuals produced seeds successfully, the number of well-developed seeds varied among species (ANOVA, *F*(2, 16) = 7.209, *p* < 0.01). While the average seed production rate for triploid *S. dolomiticola* was 1.14 seeds per fruit, this parameter decreased in both tetraploids, with *S. amici-petri* and *S. hornadensis* showing average values of 0.36 and 0.29, respectively (Figure 3). Many seeds were infected by insect larvae, and this frequency was higher in tetraploids when compared to triploids, which may be the reason for the observed pattern.

### 2.4. Flow Cytometric Seed Screen

**Figure 4:**
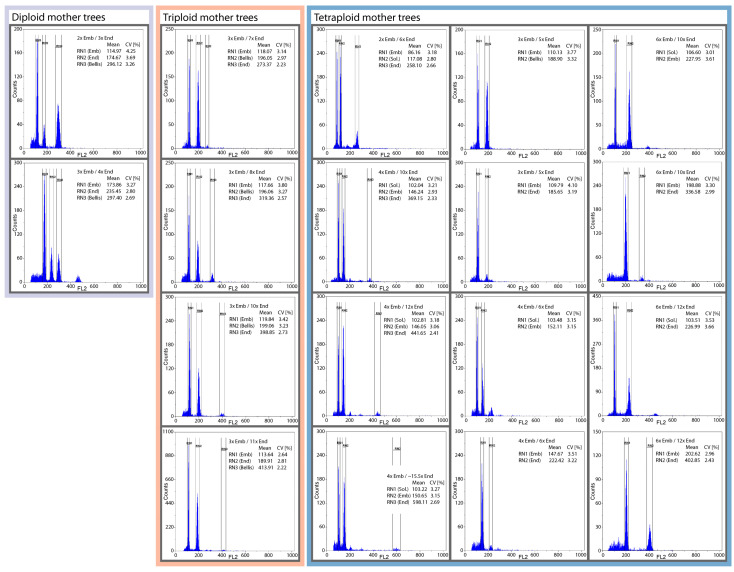
Examples of flow cytometric seed screen (FCSS) histograms and relative fluorescence (*FL2*) vs. counts of nuclei (*Counts*) for most frequent seed types in diploids, triploids, and tetraploids. Each analyzed seed is outlined in dark grey. Two separate measurements, embryo + reference standard and embryo + endosperm, were necessary to clarify the reproduction mode of some seeds. Mean value of fluorescence intensity of nuclei (*Mean*) delimited in peaks (*RN1*–*RN3*) and corresponding coefficient of variation (*CV*) are reported. *Emb*–embryo, *End*–endosperm, *Bellis*–reference standard *Bellis perennis*, *Sol.*–reference standard *Solanum lycopersicum*.

The results of the FCSS analyses showed that 8 out of 316 seeds (2.53%) had only one peak (for embryo) on FCSS histograms, which did not allow for the determination of reproduction modes in these cases. Three seeds showed more than two expected (embryo and endosperm) peaks on FCSS histograms, which suggests the origin of possible “twins”–two developed embryo sacs within a single ovule. These were not necessarily uninterpretable, but we decided to exclude them from further analyses. We retained 305 analyzed seeds for further analyses. Examples of FCSS histograms for the most frequent seed types (see below) are shown in Figure 4.

**Figure 5:**
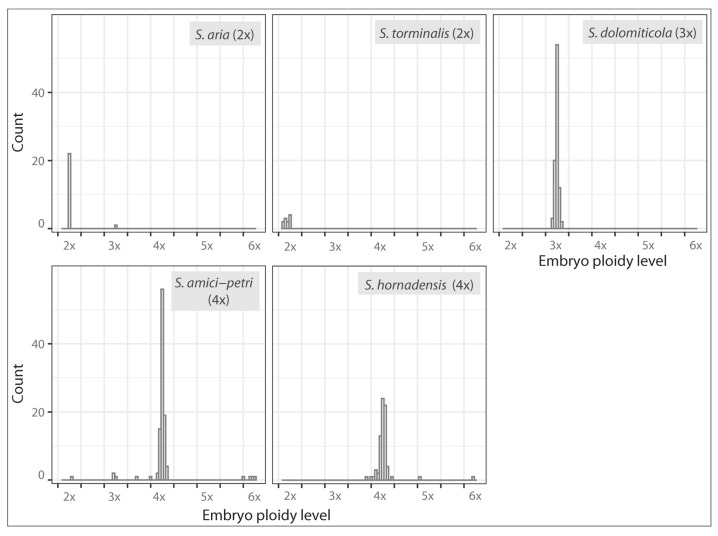
Calculated embryo ploidy levels of 305 seeds of *Sorbus* analyzed in the present study. The estimated euploid and aneuploid ploidy level categories are separated by vertical lines.

**Figure 6:**
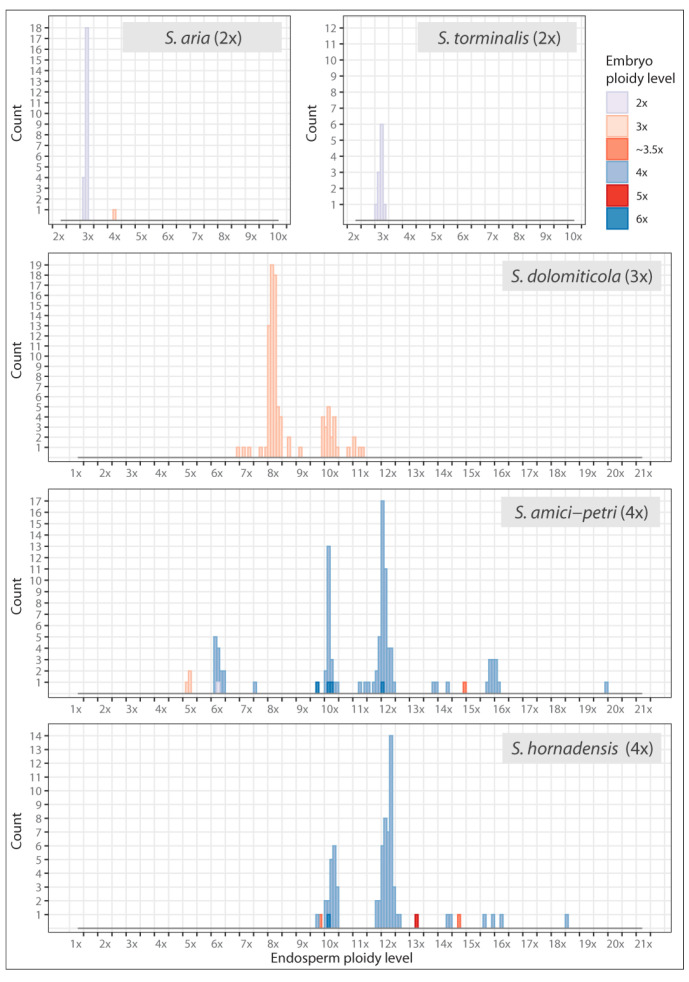
Calculated endosperm ploidy levels of 305 seeds of *Sorbus* analyzed in the present study. The estimated euploid and aneuploid ploidy level categories are separated by vertical lines. The embryo ploidy level associated with endosperm is depicted using sequential and qualitative coloring for increasing ploidy level and for even vs. odd ploidy, respectively.

Calculations of embryo ploidy levels from DNA content data enable the identification of discrete euploid categories for 303 seeds (99.34%), and 2x, 3x, 4x, 5x, and 6x embryos were found. Only two seeds were assigned to the aneuploid category of ~3.5x (Figure 5). Similarly, the ploidy levels calculated for endosperms were discrete (Figure 6), although more aneuploid categories were recorded. We identified 3x and 4x endosperms in diploids. In triploids, 8x, 10x, and 11x endosperms were most frequent, and ~6.5x, 7x, ~7.5x, ~8.5x, 9x, ~9.5x, and ~10.5x were rare. The most complex situation was documented for tetraploids. While most of the observed endosperms were 6x, 10x, 12x, ~15.5x, and 16x, we also found a variety of other ploidy levels, namely 5x, ~7.5x, ~9.5x, 11x, ~11.5x, ~12.5x, 13x, ~13.5x, 14x, ~14.5x, ~18.5x, and ~19.5x. A methodological error must be considered here: the frequency of some aneuploid categories may be slightly overestimated in the present study, as is seen, for instance, in the case of ~11.5x or ~15.5x. Calculated values are very close to neighboring euploid categories, which may stem from errors in the ploidy level identification, which increases in the cases of >12x endosperms [18,39].

### 2.5. Reproduction Modes in S. amici-petri, S. dolomiticola, and S. hornadensis

All 34 seeds analyzed in diploid *S. aria* and *S. torminalis* were found to be of sexual origin. Of these, 33 seeds were 2x_emb_/3x_end_. These can be interpreted as the result of meiotic reduced embryo sacs double-fertilized with reduced sperm cells. One 3x_emb_/4x_end_ originated from a meiotically reduced embryo sac but was double-fertilized with 2x sperm cells.

The triploid *S. dolomiticola* produced 60 (65.93%) and 16 (17.58%) exclusively asexual seeds of categories 3x_emb_/8x_end_ and 3x_emb_/10x_end_, respectively (Table 1). Embryos and endosperms of these seeds developed from apomeiotic unreduced embryo sacs (3x egg cell + 6x central cell). Embryos developed parthenogenetically from unreduced egg cells and the endosperm origin was produced via pseudogamy, indicating that central cell fertilization is necessary for the successful development of parthenogenetic embryos. The central cell (6x) was fertilized by one 2x or two 1x sperm cells (origin of 8x endosperms) or two 2x sperm cells (10x endosperms). Other seed categories were rarely represented (see Table 1). The data indicated that the species can be considered an obligate apomict.

The tetraploid *S. amici-petri* showed the highest variation in reproduction modes among the investigated endemics (Table 1). Most of the analyzed seeds were 4x, but rare 2x, 3x, ~3.5x, and 6x seeds were also recorded. The categories of 4x_emb_/10x_end_ and 4x_emb_/12x_end_ were most frequent, and 20 (18.87% in *S. amici-petri*) and 37 seeds (34.91%), respectively, were found. The embryo sacs of these seeds were apomeiotic and unreduced (4x egg cell + 8x central cell), and embryos developed parthenogenetically. The pseudogamous origin of endosperms was further inferred, with the one or two 2x sperm cells’ participation. Other seeds had 4x embryos associated with 11x, ~11.5x, ~13.5x, 14x, ~15.5x, 16x, and ~19.5 endosperms (26 seeds, 24.53%), and altogether suggested common pseudogamous apomixis in *S. amici-petri*. A considerable number of seeds (18, 16.98%) originated from meiotically reduced embryo sacs, namely 3x_emb_/5x_end_ and 4x_emb_/6x_end_ (possibly also 4x_emb_/~7.5x_end_). The only recorded seed of 2x_emb_/6x_end_ represented a rare event of reduced parthenogenesis. Finally, unreduced egg cells may also be fertilized (origin of B_III_ hybrids), as found in seed categories 6x_emb_/~9.5x_end_, 6x_emb_/10x_end_, and 6x_emb_/12x_end_.

The presence of sexually derived seeds in *S. amici-petri* has a non-random distribution across sampled trees. We found nine trees that almost exclusively produced asexually derived seeds (>80%), with only three sexual seeds out of 78 in total. The other two trees had higher proportions of sexual seeds (37.5% and 60%), with 15 sexual seeds out of 28 in total.

All endosperms in the morphologically distinct tetraploid species *S. hornadensis* showed a ploidy level higher than 8x, which indicates that the species can be considered an obligate apomict (Table 1). Most of the analyzed seeds were 4x, but ~3.5x, 5x, and 6x seeds were also occasionally recorded. The categories of 4x_emb_/10x_end_ and 4x_emb_/12x_end_ were most frequent, with 18 (24.32% in *S. hornadensis*) and 38 seeds (51.35%), respectively. The embryo sacs of these seeds were apomeiotic and unreduced (4x egg cell + 8x central cell), as in *S. amici-petri*, and the embryos developed parthenogenetically. The endosperms in these seeds were of pseudogamous origin, with an 8x central cell fertilized by one or two 2x sperm cells. Furthermore, 14 seeds (18.92%) containing 4x embryos had endosperms of various ploidy levels, namely ~9.5x, ~11.5x, ~12.5x, 13x, 14x, ~15.5x, 16x, and ~18.5x. Rare B_III_ hybrids may also appear in *S. hornadensis*, which was evidenced by the presence of two seeds of the categories 5x_emb_/13x_end_ and 6x_emb_/10x_end_. Additionally, we recorded two seeds with decreased embryo and endosperm DNA content, namely ~3.5x_emb_/~9.5x_end_ and ~3.5x_emb_/~14.5x_end_, for which the possible route for such DNA contents is difficult to infer. In both of these seeds, a possible partial reduction of DNA content may be explained by the loss of some chromosomes.

## 3. Discussion

### 3.1. Reproduction Characteristics and Variation of Tetraploid S. amici-petri and S. hornadensis

Studies of reproduction modes in apomictic plants depend on the precise ploidy level determination of the egg and the central cells (embryo sac), as well as the sperm cells (pollen grain), which interact during fertilization [40,41,42]. The direct observation of gametophyte development and the fertilization process by cyto-embryological microscopic techniques in sufficiently high numbers is very time consuming and laborious [43]. In most cases, the ploidy levels of eggs and central cells can be determined based on FCSS, thereby enabling the sexual or apomictic origin of embryos to be precisely distinguished [20,39,42]. However, the employment of FCSS is limited in the estimation of the number of sperm cells contributing to endosperm [42]. For instance, the fertilization process for the seed origin with 3x_emb_/8x_end_ could be explained either by the participation of two 1x sperm cells or one 2x sperm cell in the endosperm origin, which cannot be distinguished based on FCSS. Nevertheless, FCSS is one of the most informative techniques in cyto-embryological studies, as it enables the rapid screening of hundreds of seeds in a short time period [44].

In the present study, we applied FCSS to study the reproduction modes of the stenoendemic species *S. amici-petri*, *S. dolomiticola*, and *S. hornadensis* for the first time. We confirmed their cytotypic differentiation; while *S. amici-petri* and *S. hornadensis* are tetraploids, *S. dolomiticola* is triploid species. Both tetraploids *S. amici-petri* and *S. hornadensis* are pseudogamous apomicts that predominantly reproduce clonally, with sexual reproduction being rare (see below). In most cases, we documented 4x_emb_/10x_end_ and 4x_emb_/12x_end_ seeds. Endosperms usually originated from pseudogamy, the fertilization of unreduced central cells with reduced 2x/2x + 2x sperm cells in both species. These are likely their own pollen grains because pollen’s self-compatibility is often restored in polyploids [45] and has also been documented among the tetraploid *Sorbus* in pollination and molecular studies [17]. We documented only 18 sexually derived seeds in *S. amici-petri*. Despite its rarity, sexual reproduction may effectively modify genetic makeup and increase the evolutionary potential of apomictic species [14].

We assumed a high frequency of 2x pollen in the studied locality (the presence of many tetraploids). In our study, we documented a higher proportion of 3x_emb_/8x_end_ seeds than 3x_emb_/10x_end_ seeds in triploids, which may suggest more frequent one-sperm-fertilized central cells (1 × 2x over 2 × 2x sperm cells from tetraploids); however, the opposite pattern was observed in tetraploids (more 4x_emb_/12x_end_ than 4x_emb_/10x_end_ seeds). In the case of triploids, we could not confirm the ploidy levels of sperm cells, as both 1x pollen from diploids and 2x pollen from tetraploids may possibly be utilized in the pollination of triploid. Therefore, frequent 8x endosperms may possibly be the result of the higher pollen compatibility of triploids with 1x pollen from diploids, and in this case, the two-sperm-fertilization of the central cell is equally frequent in triploids as it is in tetraploids. In the case of tetraploids, which are likely self-compatible [17], the central cell is likely more frequently fertilized by the two sperm cells than only by the one sperm cell. Both possibilities are apparently possible. The question of the biological role of one-sperm- vs. two-sperm-fertilization of central cells has been debated. The genus *Sorbus* belongs to the subtribe Malinae Reveal within Rosaceae that includes other genera whose reproduction modes are similar to those of *Sorbus* [39,46,47,48,49]. The predominance of two sperm cells over one sperm cell in the fertilization of the central cell is typical in *Amelanchier* Medik. [47] and has been reported in other studies of *Sorbus* as well [18,20]. In contrast, the one-sperm fertilization of the central cell is more frequent in *Cotoneaster* Medik. [48,49]. For *Crataegus* L. species, both alternatives were predominant in different species [39,46,50]. Therefore, this question remains to be resolved for rosaceous genera, but reproductive modes may differ even within plants of a single genus.

*Sorbus amici-petri* and *S. hornadensis* include seeds with 4x embryos and ~16x endosperms (~15.5x, 16x). We inferred that such seeds are more frequent in *S. amici-petri*. The origin of endosperm in this case may involve trinucleated central cells (12x) fertilized with 2x + 2x sperm cells or the endopolyploidization of autonomous endosperms (8x → 16x). Trinucleated central cells have been frequently inferred in seed origins of several roseaceous genera, including *Amelanchier*, *Cotoneaster*, *Crataegus*, and *Sorbus* [18,20,39,46,47,48,49]. An alternative explanation that involves endopolyploidization has been proposed, for instance, in some triploids of *Crataegus* [50], which are obligate apomicts with 6x central cells, and rarely produce seed types of 6x_emb_/>12x_end_. The origin of such seeds may be better explained as a consequence of the endopolyploidization of unreduced embryo sacs or newly originated embryos and endosperms simultaneously. Generally, species may differ either in various pathways of embryo and endosperm origin, and even in their variation within the species.

*Sorbus amici-petri* can produce asexual as well as sexual seeds. The presence of seeds with 2x_emb_/6x_end_, 3x_emb_/5x_end_, and 4x_emb_/6x_end_ indicates rare regular meiosis in *S. amici-petri*. Most of these seeds contain embryos produced by the sperm-cell fertilization of reduced egg cells. Even if embryo sacs are of apomeiotic origin, egg cells are frequently fertilized and B_III_ hybrids originate (6x_emb_/10x_end_). We recorded almost exclusively the 2x sperm cell contribution to the endosperm origin, irrespective of origin from the fertilization of reduced or unreduced central cells. Rarely, we found that, for the 1x sperm cells contribution (3x_emb_/5x_end_), the pollen donor was likely diploid *S. aria* or *S. torminalis* (they predominate in populations). The frequency of both reproduction modes (sexual or apomictic) varied between individuals of *S. amici-petri*. Meiotically derived embryos were found on certain trees with a frequency of 0–60%. Our sample size of this local endemic does not allow for general comparison. However, some apomicts of the genus *Boechera* Á. Löve et D. Löve showed a bimodal distribution of the frequency of apomeiosis [51]. This means that facultative apomicts produced asexual seeds at very low or very high frequencies, and those with intermediate frequencies were absent. This pattern has yet to be fully explained, but is likely a result of interplay between environmental effects during gamete development, gene flow dynamics in the populations, and regulatory changes associated with apomeiosis [51].

### 3.2. Dependence of Seed Production of Apomict S. dolomiticola on Heterospecific Pollen and Conservation Implications

We have shown that *S. dolomiticola* is an apomictic species, and we have exclusively documented the unreduced embryo sac formation and parthenogenetic embryo origins. Nevertheless, sperm cells are important in the reproduction of *S. dolomiticola*. Although necessary for the origin of functional endosperms, they are excluded from egg cell fertilization, and thus do not affect the genotype of the embryo. Contributions of 2x (or 1x + 1x) and two 2x, sperm cells are the most frequent among reconstructed endosperm origins. Species of the genus *Sorbus* produce reduced pollen grains [20], as do related genera, e.g., *Crataegus* [52], and although we did not perform ploidy analyses of pollen cells, our pollen investigations revealed that *S. dolomiticola* has irregular pollen grains, which suggested an unbalanced reduced pollen grain formation [53]. However, contributions of unbalanced ~1.5x sperm cells, as manifested by ~7.5x or 9x (~9.5x) endosperms, are very rare in *S. dolomiticola*. This is consistent with results of another study [20], but opposite patterns, including the frequent fertilization of unreduced central cells of triploids by unbalanced reduced ~1.5x sperm cells (origin of 3x_emb_/9x_end_ and 3x_emb_/~9.5x_end_), were documented in *Sorbus* [18]. Additionally, this is the case for *Crataegus* L. [50,52]. The self-incompatibility observed in triploid *Sorbus* [17] may also be the reason for the low frequency of ~1.5x pollinations. Because *S. dolomiticola* is an apomictic species and forms clonal populations, pollen incompatibility is very likely effective, even among different individuals (supposedly genetically identical clones).

Our results show that contributions of heterospecific pollen from diploids (1x/1x + 1x) or tetraploids (2x/2x + 2x) is necessary for the origin of nutritive tissue endosperm (pseudogamy), and regular seed production in *S. dolomiticola*. This latter example suggests that the evolutionary success of self-incompatible or sterile pseudogamous apomicts therefore depends on other related species. This has an important implication for conservation strategies of such endemics. Seed formation in *S. dolomiticola* trees is conditioned by the sympatric occurrence of cross-compatible congeners. Mating cytotypically differentiated partners must be maintained in sympatry with *S. dolomiticola* to ensure the production of well-developed seeds and the long-term survival of the species [38]. Unless precise inter-species compatibilities are known, conservation priorities should also focus on the preservation of population heterogeneity, with the presence of several cytotypically variable *Sorbus* species [54]. Absence of congeners at sites may potentially lead to *S. dolomiticola* extinction. Several similar biological associations have implications in conservation management. Conservation efforts for rare parasitic plants, which may be limited due to host availability or host preference, should consider the needs of the host [55], and should appropriately adjust conservation management accordingly. Conservation implications should also be considered in cases of plant-insect interactions. The conservation of dependent species has been proposed for threatened herbivorous insects that depend on specific host plants [56]. The dependent species are often lost due to extinction before their host [55,56]; conservation planning should not ignore such biotic interactions.

### 3.3. Microevolutionary Dynamics in Mixed-Cytotype Populations in Stredné Pohornádie Valley

The origin of parthenogenetic 2x, allotriploid 3x, and sexual 4x individuals, as well as B_III_ 6x hybrids, represents a palette of various cytotypes with microevolutionary potential. Mature plants with the ability to produce functional pollen and seeds can interact with co-occurring species. Mixed cytotype populations of pseudogamous and facultative apomicts are, therefore, considered natural evolutionary laboratories. Cytotypic diversity in populations results in either a higher proportion of observed 3x offspring on diploid plants [18] or a larger proportion of hybrid plants. They are likely to have originated because of a higher proportion of cytotypically diverse pollen available for pollination [20,39]. For instance, either the reduced 2x pollen from 4x plants or the unreduced 2x pollen from 2x plants may give rise to 3x embryos in 2x trees. However, FCM does not allow interploidy to be distinguished from within-ploidy crossings.

The presence of various endemics of *Sorbus* in Stredné Pohornádie valley is not surprising. Heterogeneous orography and mosaics of forests, xerothermous forest-steppe-like habitats, and rocky terraces allow several *Sorbus* species to co-exist in relatively close proximity [34,35,36]. The diploids *S. aria*, *S. aucuparia*, and *S. torminalis* may be found together with several other polyploid taxa, such as *S. danubialis* s.l or *S. thaiszii* s.l. Their mutual reproductive interactions and between-cytotype crosses may result in locally originating, hybrid triploids [57] that can serve as a triploid bridge to the origin of new genotypes. Gametophytic apomixis is likely a process that can stabilize new genotypes and lead to the evolution of new species [27,45]. Stredné Pohornádie valley may be considered a region with several evolutionarily significant units, with the ongoing speciation of *Sorbus*. Presumed combinations of parental species *S. danubialis* s.l., *S. thaiszii* s.l., *S. aucuparia*, and *S. torminalis* for the hybridogenous origin of three stenoendemics were proposed based on morphological variations and they need to be tested as probable hypotheses using artificial pollination techniques and molecular and cytogenomic research tools. 

## 4. Materials and Methods

### 4.1. Plant Material 

Three species of the genus *Sorbus*: *S. amici-petri*, *S. dolomiticola*, and *S. hornadensis* (Figure 1) are narrow endemics, which are known only from a few sites in the close vicinity of the Kysak, Obišovce, and Trebejov villages (Stredné Pohornádie phytogeographical district, eastern Slovakia, Central Europe, [58]), which are located approximately 15 km north of Košice city. In this study, trees were sampled from two xerothermous steep S-W oriented slopes (N: 48°51′52″ E: 21°13′00″, alt. ~320 m a.s.l. and N: 48°50′46″, E: 21°14′10″, ~320 m a.s.l). Floral buds were collected in the spring (May) of 2022 and mature fruits in the autumn (October–November) of 2020 and 2022; all seeds originated from open-pollination. Details of the sampled material are listed in Table 2. Additionally, fruits of two diploid species, *S. aria* and *S. torminalis*, which grow sympatrically with the investigated polyploids, were sampled for comparison and the correct interpretation of flow cytometry (FCM) data. Voucher specimens of the trees were deposited in the herbarium of the Botanical Garden of Pavol Jozef Šafárik University in Košice [59].

### 4.2. Pollen Stainability and Pollen Grain Size Determination

Male meiosis may be irregular in polyploids; therefore, pollen viability and the shape of pollen grains may be indicative of the disruption of male gamete development. In the present study, pollen viability was assessed indirectly using a staining technique [60]. We used a simplified method for the differential staining of aborted and non-aborted pollen grains [61]. Floral buds in the balloon stage (larger buds close to anthesis [17]) or open flowers were sampled and immediately placed in a solution of ethanol (96%) and acetic acid (98%) in a ratio of 3:1. Four to five anthers were dissected from one randomly selected bud (or flower) per tree and placed in 60 μL of staining solution prepared according to Ross et al. [61]. After incubating for ~30 min and heating for 5 min (moving the glass slide above a flame), anthers were thoroughly crushed on a glass slide until pollen grains were released into the staining solution. Prepared slides were photographed and analyzed immediately using a Leica DM 2500 microscope equipped with a DFC 290 HD camera (Leica Microsystems GmbH, Wetzlar, Germany) and the Leica application suite software (ver. 3.5.0, Leica Microsystems GmbH, Wetzlar, Germany). The slides were then observed under 400× magnification. We scored at least 80 pollen grains per sample. The proportion of stained pollen grains was calculated as a percentage: 100 × (number of stained pollen grains)/(number of all pollen grains).

The size and shape of pollen grains were determined via image analysis using TpsDig2 software (ver. 2.26, [62]). Pollen size was scored as the area of the 2D projection of the stained pollen grain (in μm^2^).

### 4.3. Seed Production Rate

Fruits were transversely halved and seeds with well-developed embryos were counted. We expressed the seed production rate as the ratio between the number well-developed seeds (Ns) and the number of examined fruits (Nf).

### 4.4. Flow Cytometric Determination of the Ploidy Level of Mother Plant, Embryo, and Endosperm

FCM was used to determine the ploidy level of investigated mother individuals (flow cytometry ploidy level determination; FCP [63]) and their seed tissues, embryo, and endosperm (flow cytometric seed screen; FCSS [40,41]). The ploidy level was determined based on DNA content determinations of nuclei in maternal (mother DNA content/ploidy level), embryo (embryo DNA content/ploidy level), and endosperm tissue (endosperm DNA content/ploidy level). We used terminal or subterminal buds on short brachyblasts of *Sorbus* trees as a source material for nuclear isolation and subsequent FCP. Seeds were halved, and embryos and endosperms were used to isolate nuclei for FCSS. We conducted a two-step FCM procedure [64]. Samples were prepared using the following internal reference standards [65,66]: *Solanum lycopersicum* L. ‘Stupické polní tyčkové rané’, 1.96 pg DNA [67] or *Bellis perennis* L. wild population, 3.61 pg DNA [68] (note, we recalculated the genome size of *B. perennis* against *S. lycopersicum*). We used a modified sample preparation protocol [52,69]. Approximately 0.5 cm^2^ of the leaf of the reference standard and two or three buds of the *Sorbus* tree and halves of seeds were co-chopped using a razor blade in a petri dish containing 1 mL of general purpose buffer for FCP and FCSS, respectively [69]. A suspension containing isolated nuclei was then filtered through a 42-μm nylon filter. Samples were supplemented with 4 μL of β-mercaptoethanol, 50 μL RNAse (1 mg mL^−1^), and 50 μL of the intercalating dye propidium iodide (1 mg mL^−1^). After 10 min of incubation at 4 °C, the fluorescence intensity of stained nuclei was measured using a Partec CyFlow ML flow cytometer (Partec GmbH, Münster, Germany) and FloMax software (ver. 2.7, Partec GmbH, Münster, Germany). We recorded at least 4000 measured nuclei via FCM (min. 3780 but often more than 5000 nuclei), usually with at least 1000 nuclei for both the *Sorbus* and reference standard. The coefficient of variation of the nuclei was <5% in FCM peaks of *Sorbus* (maternal tissues or embryos) and the reference standard, and <8% in peaks of endosperms (mostly because of less abundant endosperms in *Sorbus* seeds).

The DNA content of mother plants was calculated based on the following formula, where a deviation of 1.1% from flow cytometry machine linearity was incorporated:

DNA quantity of sample = DNA quantity of standard × [(G_0_/G_1_ peak mean of *Sorbus*)/1.011 × (G_0_/G_1_ peak mean of standard)]

where G_0_/G_1_ refers to the population of nuclei (FCM peak) in G_0_ or G_1_ phases of the cell cycle. The DNA quantity and ploidy level determination were interpreted based on the previous inference of relatively stable and distinct DNA content differences observed between 2x, 3x, 4x, and 5x individuals [70].

In FCSS analyses, we first determined the embryo DNA content and then calculated the endosperm DNA content as follows:

DNA quantity of endosperm = DNA quantity of embryo × [(G_0_/G_1_ peak mean of endosperm)/1.011 × (G_0_/G_1_ peak mean of embryo)]

where G_0_/G_1_ refers to the population of nuclei (FCM peak) in G_0_ or G_1_ phases of the cell cycle. This is useful when separate measurements, such as the embryo + reference standard and embryo + endosperm, are performed. We determined embryo and endosperm ploidy levels based on the inference of their DNA contents, adjusted for the mean DNA content of embryos in diploids (those of the same 2x ploidy level as their mother trees averaged per mother tree) or of parthenogenetic embryos in polyploids (averaged per mother tree). We analyzed 282 seeds of triploids and tetraploids, and 34 seeds of diploids for comparison.

### 4.5. Examination of Reproduction Modes

We inferred the occurrence of meiosis/apomeiosis, the autonomous/pseudogamous endosperm formation, the embryo origin (parthenogenesis/sexual origin/B_III_ hybrids), and the contribution of sperm cells in the embryo and the endosperm origin, as per recent studies [40,41,42]. The reconstruction of the embryo and endosperm origin in seeds is crucial for the determination of reproduction modes in the FCSS method [40,41,42]. Possible interpretations of the seed origin are summarized in Supplementary Material Table S1.

### 4.6. Statistical Analyses

All summary statistics and plots were performed using the R environment (ver. 3.5.3 [71]) and the ggplot2 package [72]. One-way analysis of variance (ANOVA) and Tukey’s HSD pairwise multiple comparison test were employed to test for differences between the means of the seed production rate of different species. Data were log transformed to meet assumptions of ANOVA, data normality and homoscedasticity. 

## 5. Conclusions

In the present study, we documented the reproduction modes of triploid *S. dolomiticola* and tetraploid *S. amici-petri* and *S. hornadensis*, three stenoendemics of the Stredné Pohornádie phytogeographical district in eastern Slovakia. All three species reproduced predominantly asexually, and offspring were identical clones of mother trees. Embryos developed parthenogenetically from unreduced egg cells, but pseudogamy, the fertilization of the unreduced central cells of embryo sacs, is necessary for regular seed development. While tetraploids produced regular pollen grains, and these were involved in the pseudogamic origin of endosperm, triploid *S. dolomiticola* formed irregular pollen grains and appeared to be dependent on pollen availability from different cytotypes (diploids and tetraploids). Rare, sexually originated seeds in tetraploids, mostly in *S. amici-petri*, may increase the genetic and also cytotypic diversity of populations and may further accelerate within-population microevolutionary dynamics; this may potentially lead to the origin of new species. We conclude that determining the modes of reproduction in endangered pseudogamous apomicts may help to set up appropriate conservation strategies. Conservation planning focused on triploids, as in the case of *S. dolomiticola*, should include strategies that maintain or even increase the species and cytotypic diversity of *Sorbus* populations.

## Figures and Tables

**Table 1:** Frequency of seed categories in *Sorbus amici-petri*, *S. dolomiticola*, and *S. hornadensis*. Embryo ploidy levels are listed in rows and endosperm ploidy levels are listed in columns.

		Endosperm Ploidy Level																								
	*S. amici-petri*																									
		3x	4x	5x	6x	~6.5x	7x	~7.5x	8x	~8.5x	9x	~9.5x	10x	~10.5x	11x	~11.5x	12x	~12.5x	13x	~13.5x	14x	~14.5x	~15.5x	16x	~18.5x	~19.5x
**Embryo ploidy level**	2x	.	.	.	1	.	.	.	.	.	.	.	.	.	.	.	.	.	.	.	.	.	.	.	.	.
	3x	.	.	3	.	.	.	.	.	.	.	.	.	.	.	.	.	.	.	.	.	.	.	.	.	.
	~3.5x	.	.	.	.	.	.	.	.	.	.	.	.	.	.	.	.	.	.	.	.	1	.	.	.	.
	4x	.	.	.	13	.	.	1	.	.	.	.	20	.	2	9	37	.	.	2	1	.	7	4	.	1
	6x	.	.	.	.	.	.	.	.	.	.	1	2	.	.	.	1	.	.	.	.	.	.	.	.	.
	*S. dolomiticola*																									
		3x	4x	5x	6x	~6.5x	7x	~7.5x	8x	~8.5x	9x	~9.5x	10x	~10.5x	11x	~11.5x	12x	~12.5x	13x	~13.5x	14x	~14.5x	~15.5x	16x	~18.5x	~19.5x
	3x	.	.	.	.	1	2	1	60	2	1	3	16	1	4	.	.	.	.	.	.	.	.	.	.	.
	*S. hornadensis*																									
		3x	4x	5x	6x	~6.5x	7x	~7.5x	8x	~8.5x	9x	~9.5x	10x	~10.5x	11x	~11.5x	12x	~12.5x	13x	~13.5x	14x	~14.5x	~15.5x	16x	~18.5x	~19.5x
	~3.5x	.	.	.	.	.	.	.	.	.	.	1	.	.	.	.	.	.	.	.	.	1	.	.	.	.
	4x	.	.	.	.	.	.	.	.	.	.	1	18	.	.	4	38	2	1	.	2	.	2	1	1	.
	5x	.	.	.	.	.	.	.	.	.	.	.	.	.	.	.	.	.	1	.	.	.	.	.	.	.
	6x	.	.	.	.	.	.	.	.	.	.	.	1	.	.	.	.	.	.	.	.	.	.	.	.	.

**Table 2:** Summary of plant material belonging to the genus *Sorbus* analyzed in the present study. The number of investigated individuals and number of pollen grains, fruits, and seeds used for pollen analyses (*Pollen Stainability* and *Pollen Grain Size*), seed production rate determination (*Fruits*), flow cytometry determination of ploidy level of mature plants (*FCP*), and flow cytometric seed screen (*FCSS*) are reported in parenthesis. For FCSS, the number of analyzed seeds and number of successfully analyzed seeds are mentioned before and after the forward slash, respectively.

Species	Pollen Stainability	Pollen Grain Size	Fruits	FCP	FCSS
*Sorbus amici-petri*	2 (401)	2 (192)	8 (327)	15	11 (107/106)
*S. dolomiticola*	3 (550)	3 (246)	4 (46)	15	9 (93/91)
*S. hornadensis*	3 (409)	3 (159)	7 (176)	12	10 (82/74)
SUM	8 (1360)	8 (597)	19 (549)	42	30 (282/271)

## Data Availability

Not applicable.

## Supplementary Materials

The following supporting information can be downloaded at: https://www.mdpi.com/article/10.3390/plants12020373/s1, Table S1: Survey of seed types recorded in the present study.
