# Supplementary material for: Reproduction Modes and Conservation Implications in Three Polyploid Sorbus Stenoendemics in Eastern Slovakia (Central Europe)
Source: Plants (Basel). 2023 Jan 13;12(2):373. doi: 10.3390/plants12020373 (PMC9863969; doi:10.3390/plants12020373)
Supplement: Supplementary file 1 [file plants-12-00373-s001.zip › plants-2101811-supplementary.pdf]

Supplementary Material Table S1

Survey of seed types recorded in the present study. Ploidy level of mother trees (*Mother*), number of seeds (*Seeds*), inferred ploidy level of embryo (*Embryo*), endosperm (*Endosperm*), egg (*Egg Cell*), and central (*Central Cell*) cells are reported. Embryo (*Embryo Origin*) and endosperm (*Endosperm Origin*) origins are interpreted based on inferred ploidy level of embryo and endosperm. Alternative interpretations are separated with slash mark „/“. Sexual or apomictic seed origin (*Seed Origin*) is indicated. If necessary, explanations are given in a note (*Note*). Unbalanced ploidy level of sperm cells, embryos, and endosperms are marked with „~“. Note that estimation of ploidy level is not precise in the cases of >12x endosperms, because measurement error is higher with increasing ploidy level.

Mother	Seeds	Embryo	Endosperm	Egg cell	Central Cell	Embryo origin ((Egg Cell) + Sperm Cells)	Endosperm Origin ((Central Cell) + Sperm Cell)	Seed origin	Note
2x	33	2x	3x	1x	2x	(1x) + 1x	(2x) + 1x	sexual	
2x	1	3x	4x	1x	2x	(1x) + 2x	(2x) + 2x	sexual	
3x	1	3x	~6.5x	3x	6x	(3x) + 0	(6x) + ~0.5x	apomictic	
3x	2	3x	7x	3x	6x	(3x) + 0	(6x) + 1x	apomictic	
3x	1	3x	~7.5x	3x	6x	(3x) + 0	(6x) + ~1.5x	apomictic	
3x	60	3x	8x	3x	6x	(3x) + 0	(6x) + 1x + 1x / (6x) + 2x	apomictic	
3x	2	3x	~8.5x	3x	6x	(3x) + 0	(6x) + ~1.5x + ~1.5x / (6x) + ~2.5x	apomictic	
3x	1	3x	9x	3x	6x	(3x) + 0	(6x) + ~1.5x + ~1.5x / (6x) + 3x	apomictic	
3x	3	3x	~9.5x	3x	6x	(3x) + 0	(6x) + ~1.5x + ~1.5x	apomictic	
3x	16	3x	10x	3x	6x	(3x) + 0	(6x) + 2x + 2x	apomictic	
3x	1	3x	~10.5x	3x	6x	(3x) + 0	(6x) + ~2.5x + ~2.5x	apomictic	
3x	4	3x	11x	3x	6x	(3x) + 0	(6x) + ~2.5x + ~2.5x	apomictic	

Mother	Seeds	Embryo	Endosperm	Egg cell	Central Cell	Embryo origin ((Egg Cell) + Sperm Cells)	Endosperm Origin ((Central Cell) + Sperm Cell)	Seed origin	Note
4x	1	2x	6x	2x	4x	(2x) + 0	(4x) + 1x + 1x / (4x) + 2x	apomictic	
4x	3	3x	5x	2x	4x	(2x) + 1x	(4x) + 1x	sexual	
4x	1	~3.5x	~9.5x	~3.5x	7x	(~3.5x) + 0	not interpreted	apomictic	reduced genome size of embryo compared to other 4x embryos
4x	1	~3.5x	~14.5x	~3.5x	7x	(~3.5x) + 0	not interpreted	apomictic	high CV in FCSS measurements, possibly 4x embryo
4x	13	4x	6x	2x	4x	(2x) + 2x	(4x) + 2x	sexual	
4x	1	4x	~7.5x	2x	4x	(2x) + 2x	(4x) + 2x + ~1.5x	sexual	possibly polyspermy
4x	1	4x	~9.5x	4x	8x	(4x) + 0	(8x) + ~1.5x	apomictic	
4x	38	4x	10x	4x	8x	(4x) + 0	(8x) + 2x	apomictic	
4x	2	4x	11x	4x	8x	(4x) + 0	(8x) + ~1.5x + ~1.5x	apomictic	
4x	13	4x	~11.5x	4x	8x	(4x) + 0	(8x) + ~1.5x + ~1.5x	apomictic	some of these endosperms may be 12x, because slightly underestimated genome size of endosperm
4x	75	4x	12x	4x	8x	(4x) + 0	(8x) + 2x + 2x	apomictic	
4x	2	4x	~12.5x	4x	8x	(4x) + 0	(8x) + 2x + 2x	apomictic	these endosperms may be 12x, because slightly overerestimated genome size of endosperm
4x	1	4x	13x	4x	8x / 12x	(4x) + 0	(8x) + ~2.5x + ~2.5x / (12x) + 1x	apomictic	possibly trinucleated central cell
4x	1	4x	~13.5x	4x	8x / 12x	(4x) + 0	(8x) + ~2.5x + ~2.5x / (12x) + ~1.5x	apomictic	possibly trinucleated central cell
4x	3	4x	14x	4x	12x	(4x) + 0	(12x) + 2x	apomictic	possibly trinucleated central cell

Mother	Seeds	Embryo	Endosperm	Egg cell	Central Cell	Embryo origin ((Egg Cell) + Sperm Cells)	Endosperm Origin ((Central Cell) + Sperm Cell)	Seed origin	Note
4x	2	4x	~14.5x	4x	12x	(4x) + 0	(12x) + ~2.5x	apomictic	possibly trinucleated central cell
4x	9	4x	~15.5x	4x	8x / 12x	(4x) + 0	(2×8x) + 0 / (12x) + 2x + 2x	apomictic	these endosperms may be 16x, because slightly underestimated genome size of endosperm; possibly trinucleated central cell or endopolyploidization of endosperm
4x	5	4x	16x	4x	8x / 12x	(4x) + 0	(2×8x) + 0 / (12x) + 2x + 2x	apomictic	possibly trinucleated central cell or endopolyploidization of endosperm
4x	1	4x	~18.5x	4x	8x / 16x	(4x) + 0	(2×8x) + ~1.5x + ~1.5x / (12x) + 2x + 2x	apomictic	possibly tetranucleated central cell or endopolyploidization of endosperm
4x	1	4x	~19.5x	4x	8x / 16x	(4x) + 0	(2×8x) + ~1.5x + ~1.5x / (12x) + 2x + 2x	apomictic	these endosperms may be 20x, because slightly underestimated genome size of endosperm; possibly tetranucleated central cell or endopolyploidization of endosperm
4x	1	5x	13x	4x	12x	(4x) + 1	(12x) + 1x	apomictic	B_{III} hybrid, possibly trinucleated central cell
4x	1	6x	~9.5x	4x	8x	(4x) + 2	(8x) + ~1.5x	apomictic	B_{III} hybrid
4x	3	6x	10x	4x	8x	(4x) + 2	(8x) + 2x	apomictic	B_{III} hybrid
4x	1	6x	12x	4x	8x / 12x	(4x) + 2	(8x) + 2x + 2x / (12x) + 0	apomictic	B_{III} hybrid, polyspermy or trinucleated central cell
